# ZnO NPs: A Nanomaterial-Based Fertilizer That Significantly Enhanced Salt Tolerance of *Glycyrrhiza uralensis* Fisch and Improved the Yield and Quality of Its Root

**DOI:** 10.3390/plants14121763

**Published:** 2025-06-09

**Authors:** Ning Wu, Miao Ma

**Affiliations:** Ministry of Education Key Laboratory of Xinjiang Phytomedicine Resource Utilization, The Key Laboratory of Oasis Town and Mountain-Basin System Ecology, College of Life Sciences, Shihezi University, Shihezi 832003, China; 20222006043@stu.shzu.edu.cn

**Keywords:** herb, licorice, physiological mechanism, salinity, ZnO nanoparticle

## Abstract

*Glycyrrhiza uralensis* Fisch. is an important economic plant. With its wild populations on the brink of extinction and the area of salinized soil increasing sharply, farmers have gradually used saline soil to carry out artificial cultivation of the licorice. However, the salt stress has led to a significant decrease in the yield and quality of its medicinal organ (root), seriously restricting the sustainable development of the licorice industry. Therefore, we investigated zinc oxide nanoparticles (ZnO NPs) as a nano-fertilizer to enhance root biomass and bioactive compound accumulation under salinity. Our results indicate that under 160 mM NaCl stress, the application of 30 mg/kg ZnO NPs increased the root biomass of the licorice and the contents of glycyrrhizic acid, glycyrrhizin, and total flavonoids in the roots by 182%, 158%, 87%, and 201%, respectively. And the ZnO treatment made the enzyme activities of SOD, CAT, and POD exhibit increase, and made the levels of superoxide anions, electrolyte leakage, soluble sugar, and proline reduce. These results demonstrate that ZnO NPs not only enhance salt tolerance but also redirect metabolic resources toward medicinal compound biosynthesis. Our findings provide a mechanistic basis for utilizing nanotechnology to sustainably cultivate the licorice in marginal saline environments, bridging agricultural productivity and pharmacological value.

## 1. Introduction

According to the Food and Agriculture Organization (FAO) of the United Nations, approximately 1.5 million hectares of agricultural land worldwide are deserted annually due to salinization [1]. Soil salinization is a major impediment to improving crop productivity globally. Prolonged and intense salt stress may induce programmed cell death [2] and may even lead to the wilting of entire plants. The surface layer of salinized soil typically contains a high concentration of soluble salts, primarily composed of Na^+^ and Cl^−^ [3]. The increased osmotic pressure of the soil induces osmotic stress in plants, inhibiting their growth, and triggering the accumulation of excessive reactive oxygen species (ROS), which in turn causes oxidative stress, disrupts metabolic processes, and severely impacts both crop yield and quality, resulting in substantial economic losses to agriculture [4]. Consequently, improving the salt tolerance of crops is of paramount importance for the sustainable utilization of saline lands.

*G. uralensis* (*Glycyrrhiza uralensis* Fisch.) is a perennial herb belonging to the Leguminosae family [5]. Its dried roots contain a variety of bioactive compounds, such as glycyrrhizic acid, glycyrrhetinic acid flavonoids, etc. These constituents exhibit a range of pharmacological activities, including anti-inflammatory [6], antiviral [7], and anticancer properties, particularly the inhibition of cancer cell proliferation [8,9]. *G. uralensis* has long been utilized as a traditional medicinal herb [10,11]. The sweetness of glycyrrhizic acid and glycyrrhetinic acid is reported to be 150 and 300 times that of sucrose, respectively [12], while the sweetness of compound glycyrrhetic acid 3-O-beta-D-monoglucuronide, synthesized from glycyrrhetic acid, is more than 900 times that of sucrose [13]. Consequently, the root and its extracts of *G. uralensis* are frequently utilized as natural sweeteners in food production in various countries. Furthermore, due to the plant’s high protein content, the stems and leaves of the licorice serve as excellent forage [14]. However, overexploitation has led to the severe depletion of wild *G. uralensis*, with most of its natural populations having become extinct. Consequently, cultivated *G. uralensis* is increasingly replacing its wild sources [15]. Although its adult individuals exhibit a higher degree of tolerance to soil salinity [16], seedlings are sensitive to the salt, often suffering from root rot and mortality in salinized habitats [17], which significantly hampers its cultivation potential in saline regions. To improve the salt tolerance of licorice seedlings, extensive research has been conducted in recent years. A growing body of evidence indicated that the application of exogenous substances to improve salt tolerance is one of the most efficient strategies for addressing this issue [17,18,19]. Current innovative cultivation strategies for *G. uralensis* present notable limitations: exogenous rare earth element applications (e.g., lanthanum) induce progressive soil accumulation despite low plant uptake [20], posing chronic risks to environmental integrity and phytotoxicity. Alternative approaches, including ethanol [21] and betaine [11] supplementation, demonstrate suboptimal efficacy in enhancing licorice yield and phytochemical quality. In contrast, micronutrient-based interventions may present a sustainable alternative. These elements serve dual significance as essential cofactors for plant physiological processes and micronutrients in human metabolic pathways, offering a biocompatible strategy to reconcile agricultural productivity with ecosystem safety.

To address the challenges posed by the degradation of agricultural soil, a range of technological innovations have emerged, including improvements of fertilizer, the development of high-yield crop varieties, and the introduction of genetically modified organisms (GMOs) [22]. However, the process of developing new crop varieties is time-consuming, and the safety of GMOs remains a subject of ongoing debate. Consequently, the enhancement of conventional fertilizers has become the preferred strategy to mitigate environmental stress and improve both crop’s yield and quality. With the ongoing advancements in nanotechnology, a variety of nanoparticle-based fertilizers [23,24] have been developed, offering promising solutions for enhancing crop productivity under stress conditions. Zinc oxide (ZnO) is a conventional form of zinc fertilizer [25]. Due to their unique surface area, nanoscale size (typically < 100 nm), and high dissolution rate, ZnO nanoparticles (ZnO NPs) enhance zinc bioavailability in the soil and increase the efficiency of zinc uptake by plants. ZnO NPs are known to enhance crop productivity by improving plant resilience to various environmental stresses [26,27,28], and they have demonstrated remarkable efficacy in boosting plant salt tolerance [29]. Prior evidence empirically established ZnO NPs’ superior efficacy in enhancing salt tolerance—exceeding alternative nanomaterials like single-walled carbon nanohorns—in *S. alopecuroides* (*Sophora alopecuroides* L.) under controlled conditions [29]. Concurrently, their lower environmental risk profile at optimized doses [30] motivated our methodological selection for investigating salinity resilience in *Glycyrrhiza uralensis* Fisch., a novel model system for nanoparticle-mediated phytochemical enhancement.

However, no prior work has leveraged nanotechnology to co-optimize stress resilience, yield, and quality in the licorice under saline cultivation. In this study, the resource allocation of licorice under ZnO NPs salt stress was discussed from the aspects of growth, physiology, and metabolism, as well as root growth and the accumulation of medicinal active substances. The multiple effects of ZnO NPs on plant stress resistance, yield, and quality were systematically expounded, which brought new possibilities for improving the productivity of saline–alkali soil and reflected the novelty of this study. This research aims to provide a scientific foundation for the high-yield-and-high-quality cultivation of *G. uralensis* in saline soil.

Adult *G. uralensis* exhibits high level of salt tolerance and can adapt to salinized farmland, but excessive salt stress markedly reduces young plant root yield and the concentration of key medicinal compounds, leading to diminished quality in cultivated *G. uralensis*. Therefore, our objective is to identify an innovative approach that not only substantially promotes the root growth but also enhances concentration of the bioactive compounds of the licorice under salt stress, thereby improving both the yield and quality of the medicinal plant. In this study, the optimal dosage of ZnO NPs was determined, while elucidating the physiological mechanisms underlying improvements in quality and yield.

## 2. Materials and Methods

### 2.1. Test Instruments

Root scanner (Expression 11000XL, Epson, Suwa City, Japan), Solarbio assay kits (Beijing Solarbio Science & Technology Co., Ltd., Beijing, China), UV-Vis spectrophotometer (UV-1900, Shimadzu Corporation, Kyoto, Japan), conductivity meter (Bante 5, Shanghai Bante Instrument Co., Ltd., Shanghai, China), Jingmei assay kit (Jiangsu Jingmei Biotechnology Co., Ltd., Yancheng, China), high-performance liquid chromatography system (Agilent 1200, Agilent Technologies, Inc., Santa Clara, CA, USA), ultrasonic extractor (KQ-300E, Kunshan Ultrasonic Instrument Co., Ltd., Kunshan, China).

### 2.2. Plant Material and Experimental Design

The *G. uralensis* seeds were provided by the Institute of Licorice in Shihezi University. The experiment was conducted at the Ministry of Education Key Laboratory of Xinjiang Phytomedicine Resource Utilization at Shihezi University campus (44°31′47″ N, 86°06′28″ E) from April to October 2023. Healthy and uniform-sized seeds were selected and immersed in a 98% sulfuric acid solution for 30 min at 25 °C, then thoroughly rinsed with tap water until no sulfuric acid residue remained on the seed surface. The seeds were subsequently soaked in distilled water for 24 h. They were then evenly sown in plastic pots filled with sandy soil (bottom diameter × top diameter × height = 20 cm × 30 cm × 20 cm). Each pot contained approximately 8 kg of soil, with a 7:3 ratio of river sand to loam. A total of 15 seeds were sown per pot at a depth of 1 cm. The soil characteristics are provided in Table 1.

The fertilization regimen followed the field application scheme for *G. uralensis* [31], consisting of urea (N ≥ 46%) at 14.99 g/m^2^, calcium superphosphate (P_2_O_5_ ≥ 46%) at 23.99 g/m^2^, and potassium sulfate (K_2_O ≥ 50%) at 10.49 g/m^2^. Calcium superphosphate was uniformly incorporated into the sandy soil, while the remaining two fertilizers were applied during the seedling stage. Upon the emergence of the sixth true leaf, four seedlings with uniform growth pattern were retained per pot.

ZnO nanoparticles (particle size 30–80 nm, purity 99%, specific surface area 21.5 m^2^/g) purchased from Jiangsu Xianfeng Company (Jiangsu XFNANO Materials Tech Co., Ltd., Nanjing, China), were thoroughly mixed with sandy soil at concentrations of 10, 15, 20, 30, and 40 mg/L. Each pot was irrigated biweekly with 200 mL of 160 mM NaCl solution [19], administered a total of five times. The control group received an equivalent volume of distilled water. Detailed information for each treatment group is provided in Table 2. Each treatment was replicated three times, and a total of 21 pots were set up. On the seventh day following the final NaCl application, physiological parameters of the third fully expanded leaf from the apex of each seedling were measured. After 14 days of continuous cultivation, the roots and shoots were harvested. Plant height, stem base diameter, root morphology, biomass, and the contents of medicinal compounds in the roots were determined.

### 2.3. Measured Parameters

#### 2.3.1. Plant Growth Parameters

Height (H): The plant height was measured from the soil surface to the apical bud of each plant. For each treatment, three plants were randomly selected, and the average value was calculated.

Basal diameter (BD): The stem diameter at the soil surface was measured for each plant. Three plants were randomly selected from each treatment group, and the mean value was calculated.

Root diameter (RD): The diameter of the primary root was measured at its thickest part where the root connects to the stem. For each treatment, three plants were randomly sampled, and the average value was determined.

Root morphological indices: The root system was gently rinsed with water to remove adhering sand and soil, and it was then placed in a scanning tray (25 cm × 15 cm) of a root scanner. The roots were scanned using Epson Scan software (WinRHIZO Pro 2013a, Regent Instruments Inc., Quebec City, QC, Canada). After acquiring the root scanning images, the root analysis system WinRHIZO was used to measure root morphological parameters, including total root length (TRL), root average diameter (RAD), total root surface area (TRSA), and total root volume (TRV). For each treatment, three plants were randomly selected, and the average values of these parameters were calculated.

Shoot dry weight and root dry weight (SDW and RDW): For each treatment, three individual plants were randomly selected. The plants were separated into shoots and roots, which were then placed in paper bags and dried in an oven at 105 °C for 30 min, followed by continuous drying at 60 °C until a constant weight was achieved. The biomass (dry weight) was measured, and the average biomass of the three individuals in each treatment was calculated.

#### 2.3.2. Enzyme Activity

The activities of superoxide dismutase (SOD), peroxidase (POD), catalase (CAT), and carbonic anhydrase (CA) in *G. uralensis* leaves were determined using Solarbio assay kits. Fresh leaf samples (0.100 g) were homogenized in an extraction buffer using a mortar and pestle on ice. The homogenate was centrifuged at 8000 rpm for 10 min at 4 °C, and the supernatant was collected for subsequent analysis. Enzyme activities of SOD, POD, CAT, and CA in the supernatant were determined using a UV-Vis spectrophotometer at wavelengths of 560 nm, 470 nm, 240 nm, and 405 nm, respectively. Each treatment was measured in triplicate, and the mean enzyme activities were calculated.

#### 2.3.3. Determination of Superoxide Anion (O_2_^−.^) and Malondialdehyde Content, Electrolyte Leakage Rate and Membrane Stability Index

Superoxide anion concentration (SA) (O_2_^−.^): The superoxide anion content in plant tissues was determined using a Solarbio assay kit. Fresh leaf samples (0.100 g) were homogenized with 1 mL of extraction buffer and thoroughly ground. The homogenate was centrifuged at 12,000 rpm for 20 min at 4 °C, and the supernatant was collected for analysis. The superoxide anion concentration was measured at 530 nm using a UV-Vis spectrophotometer. Each treatment was analyzed in triplicate, and the mean value was calculated.

Electrolyte leakage (EL) and membrane stability index (MSI): The electrolyte leakage (EL) of *G. uralensis* leaves was determined using the conductivity method [32]. Fresh leaf samples (1.000 g) were placed in 15 mL centrifuge tubes containing 10 mL of deionized water and incubated at room temperature for 12 h. The conductivity of the solution (E1) was measured using a conductivity meter. The samples were then incubated in a water bath at 100 °C for 30 min. After cooling to room temperature, the conductivity (E2) was measured again. The formula of EL is EL = E1/E2 × 100%, the formula of MSI is MSI = 1 − (E1/E2) × 100%. Each treatment was measured three times, and the average value was calculated.

Malondialdehyde (MDA) concentration: The thiobarbituric acid (TBA) method was employed for determination of the MDA concentration in the leaves [33]. Fresh-leaf sample (0.500 g) was homogenized with 2 mL of 5% trichloroacetic acid (TCA) and a small amount of quartz sand. An additional 8 mL of TCA was added, and the homogenate was centrifuged at 5000 rpm for 10 min. The supernatant was mixed with 2 mL of 0.67% TBA solution and incubated in a boiling water bath for 20 min. After cooling to room temperature, the solution was centrifuged again at 5000 rpm for 10 min. The absorbance of the supernatant was measured at 450 nm, 532 nm, and 600 nm using the UV-Vis spectrophotometer. The MDA concentration was calculated using the following formula:

MDA (μmol/g FW) = 6.45 (A_532_ − A_600_) − 0.56A_450_

Each treatment was measured three times, and the average value was calculated.

#### 2.3.4. Soluble Protein, Soluble Sugar, and Proline Assay

The soluble protein (SP) content in leaves was determined using a Jingmei assay kit (double antibody sandwich method). A standard solution (80 μg/mL) was used to prepare a series of standard solutions with concentrations of 0, 2.5, 5, 10, 20, and 40 μg/mL. Fresh leaf samples (1.000 g) were homogenized in 1 mL of phosphate-buffered saline (PBS) and centrifuged at 5000 rpm for 15 min. The supernatant was transferred to a 96-well plate, and the absorbance was measured at 450 nm using a microplate reader (1530, Life Technologies Holdings Pte Ltd., Singapore). The standard curve equation was y = 0.0484x + 0.0307 (R^2^ = 0.9996), where x represents the soluble protein concentration (μg/mL). The soluble protein content in leaves was calculated using the following formula, and each treatment was measured in triplicate to obtain the mean value.

SP (μg/g FW) = x ÷ W (W is the fresh weight of the samples, g)

The soluble sugar (SS) concentration in leaves was determined using a Solarbio kit (anthrone colorimetric method). A standard solution was prepared by dissolving 10 mg of standard powder in 1 mL of distilled water to obtain a 10 mg/mL solution, which was then diluted to concentrations of 0.3, 0.2, 0.1, 0.05, 0.025, and 0.0125 mg/mL. Fresh leaf samples (0.200 g) were homogenized in 1 mL of distilled water, transferred to a 1.5 mL centrifuge tube, and incubated in a boiling water bath for 10 min. After cooling to room temperature, the homogenate was centrifuged at 8000 rpm for 10 min at room temperature. The supernatant was transferred to a 10 mL test tube and diluted to 10 mL with distilled water. The absorbance was measured at 620 nm using the microplate reader. The standard curve equation was y = 0.7386x − 0.0091 (R^2^ = 0.9984), where x represents the soluble sugar concentration (mg/mL). The soluble sugar concentration in leaves was calculated using the following formula, and each treatment was measured in triplicate to obtain the mean value.

SS (mg/g FW) = 10 × x ÷ W (W is the fresh weight of the samples, g)

The proline (Pro) concentration in leaves was determined using the acidic ninhydrin method [34]. A standard solution was prepared by dissolving 10 mg of proline in distilled water to obtain a 10 μg/mL solution, which was then diluted to concentrations of 0, 2, 4, 6, 8, and 10 μg/mL. Fresh leaf samples (0.500 g) were homogenized with a small amount of quartz sand, and the homogenate was transferred to a 10 mL centrifuge tube. The volume was adjusted to 10 mL with 80% ethanol, and the mixture was incubated in an 80 °C water bath for 20 min. After centrifugation at 3000 rpm for 10 min, the supernatant was collected. The absorbance was measured at 520 nm using the UV-Vis spectrophotometer. The standard curve equation was y = 0.003x + 0.0352 (R^2^ = 0.9997), where x represents the proline concentration (μg/mL). The proline concentration in leaves was calculated using the following formula, and each treatment was measured in triplicate to obtain the mean value.

Pro (μg/g FW) = (x × V_1_) ÷ (W × V_2_)

W is the fresh weight of the samples, g; V_1_ is total extract amount, mL; V_2_ is the used extract amount, mL

#### 2.3.5. Medicinal Ingredients Content Assay

Standard solutions of liquiritin (Li) and glycyrrhizic acid (GA) were prepared by dissolving 0.010 g of each standard in methanol to obtain stock solutions of 2000 μg/mL. These stock solutions were then serially diluted to concentrations of 1, 10, 25, 50, 100, 250, 500, and 1000 μg/mL. These solutions were analyzed using a high-performance liquid chromatography system. The absorption peaks of Li and GA were detected at wavelengths of 276 nm and 254 nm, respectively. The peak areas were calculated, and regression equations correlating concentration with peak area were established (Table 3).

Determination of the concentration of Li and GA (μg/g dry weight): Dried *G. uralensis* roots were ground into a fine powder and passed through an 80-mesh sieve, 1.000 g of the root powder was weighed precisely and extracted with 10 mL of methanol using an ultrasonic extractor at room temperature for 1 h. The extract was centrifuged at 12,000 rpm for 30 min, and the supernatant was filtered through a 0.25 μm membrane to obtain the sample solution. The concentrations of Li and GA in the sample solution were determined by a high-performance liquid chromatography (HPLC).

Concentration of total flavonoid (TF) (μg/g, dry weight): Precisely 0.005 g of liquiritin standard was weighed and dissolved in 3 mL of methanol. The solution was then diluted to prepare standard solutions according to the method described by Guo et al. [35]. The absorbance of the solutions was measured at 334 nm using the UV-Vis spectrophotometer [36], and a regression equation correlating concentration with absorbance was established: y = 0.0755x + 0.2176 (R^2^ = 0.9996).

For sample preparation, 0.500 g of powdered sample was placed in a 10 mL centrifuge tube, mixed with 5 mL of methanol, and subjected to ultrasonic extraction for 70 min. The mixture was centrifuged at 5000 rpm for 10 min, and the supernatant was collected as the sample solution. The absorbance of the sample solution was measured at 334 nm, and the total flavonoid concentration in the roots was calculated using the regression equation.

The average content of glycyrrhizic acid, liquiritin, and total flavonoids per plant (mg/plant) was calculated by multiplying the concentration of each active compound by the average root biomass per plant. Each treatment was measured in triplicate, and the mean values were calculated.

### 2.4. Data Analysis

Each parameter was measured three times, and the average value was calculated. Statistical analysis was performed using SPSS 23.0 software (IBM Corp., Armonk, NY, USA). One-way analysis of variance (ANOVA) with least significant difference (LSD) test and Duncan’s multiple range test were conducted to determine significant differences in various parameters of *G. uralensis* among different treatments. Principal component analysis (PCA) was performed to comprehensively evaluate the treatments based on the measured parameters. Figures and correlation heatmaps were generated using Origin 2024b (Origin Lab, OriginLab Corporation, Northampton, MA, USA).

## 3. Results

### 3.1. Effects of ZnO NPs on the Growth of G. uralensis Under NaCl Stress

**Figure 1:**
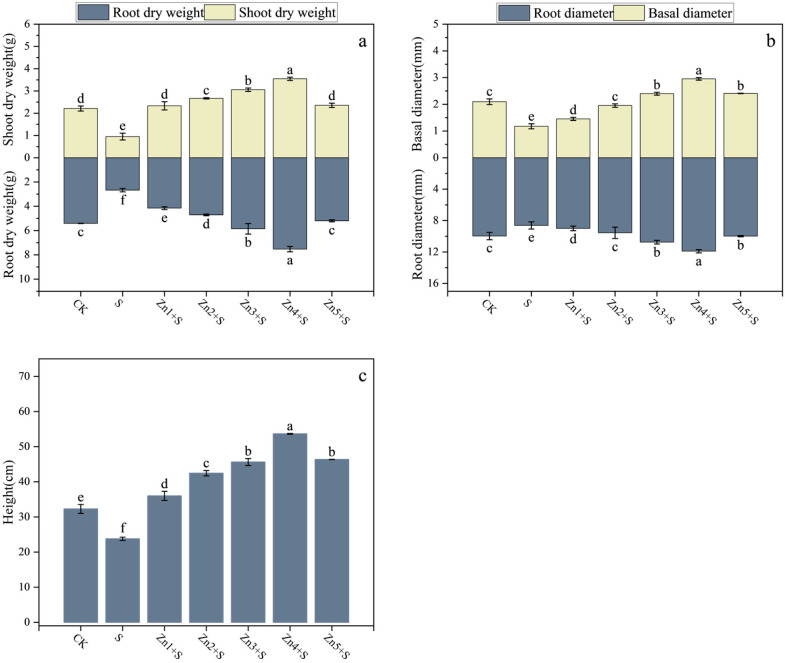
Effects of ZnO NPs application on the growth of *G. uralensis* under NaCl stress. (**a**) Shoot and root biomass, (**b**) stem basal diameter and root diameter, (**c**) plant height. Bars represent mean ± SD (n = 3). Different lowercase letters indicate statistically significant differences among treatments at *p* < 0.05 according to Duncan’s multiple range test.

**Figure 2:**
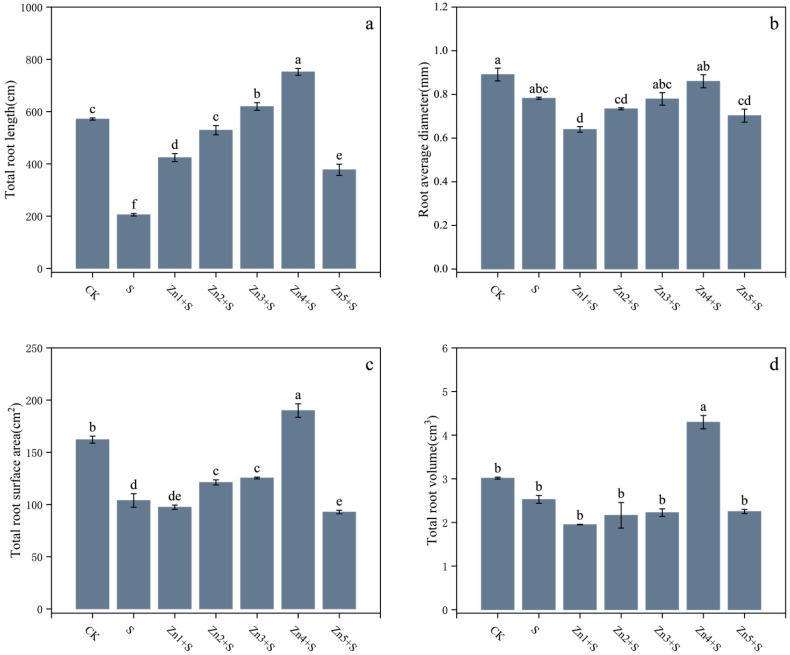
Effects of ZnO NPs application on root system development in *G. uralensis* under NaCl stress. (**a**) Total root length (TRL), (**b**) average root diameter (ARD), (**c**) total root surface area (TRSA), (**d**) total root volume (TRV). Bars represent mean ± SD (n = 3). Different lowercase letters indicate statistically significant differences among treatments at *p* < 0.05 according to Duncan’s multiple range test.

Compared to the CK, the NaCl treatment (S treatment group) resulted in reductions of 57%, 51%, 44%, 14%, 26%, 64%, 12%, 36%, and 16% in SDW, RDW, BD, RD, H, TRL, RAD, TRSA, and TRV, respectively (Figure 1 and Figure 2). The application of ZnO NPs significantly alleviated the NaCl-induced growth inhibition and promoted the growth and biomass of various organs. Among the treatments, the application of 30 mg/kg ZnO NPs (Zn4+S) showed the most pronounced ameliorative effects. Compared to the S treatment group, the Zn4+S treatment group exhibited increases of 273%, 182%, 151%, 38%, 126%, 266%, 10%, 83%, and 70% in the aforementioned parameters, respectively. Notably, even under 160 mM NaCl stress, the addition of 30 mg/kg ZnO NPs resulted in superior growth parameters compared to those under non-saline conditions (CK).

### 3.2. Effects of ZnO NPs on Enzymes Activity of G. uralensis

**Figure 3:**
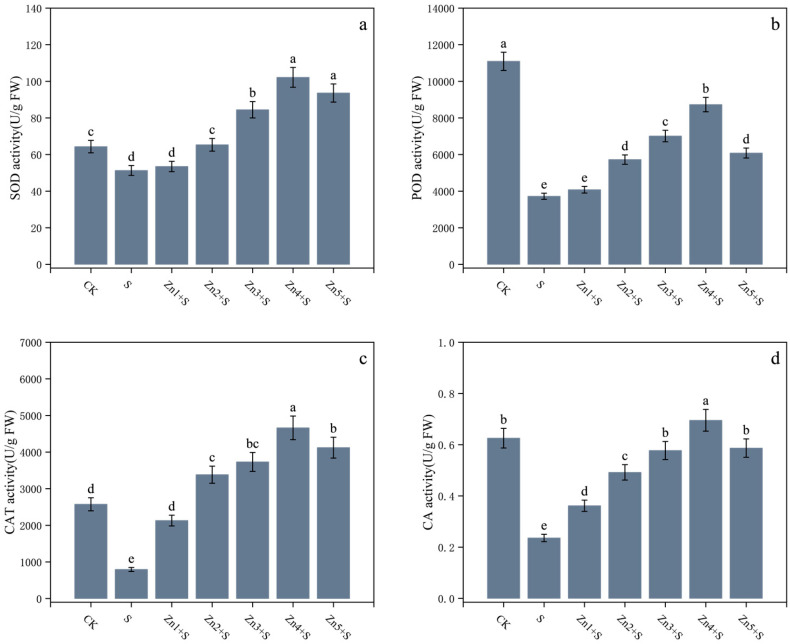
Effects of ZnO NPs application on enzymatic activities in leaves of *G. uralensis* under NaCl stress. (**a**) Superoxide dismutase (SOD) activity, (**b**) peroxidase (POD) activity, (**c**) catalase (CAT) activity, and (**d**) carbonic anhydrase (CA) activity. FW denotes fresh weight basis. Bars represent mean ± SD (n = 3). Different lowercase letters indicate statistically significant differences among treatments at *p* < 0.05 according to Duncan’s multiple range test.

Compared to the CK, NaCl application alone (S treatment) strongly inhibited the antioxidant capacity of *G. uralensis* (Figure 3). The activities of SOD, POD, CAT, and CA in its leaves decreased by 20%, 66%, 69%, and 62%, respectively. However, exogenous ZnO NPs markedly enhanced the activities of these enzymes under NaCl stress. Specifically, the Zn4+S treatment group (with 30 mg/kg ZnO NPs) showed increases of 99%, 135%, 487%, and 195%, respectively, compared to those under the S treatment (Figure 3a–d). Notably, the SOD (Figure 3a) and CAT (Figure 3c) activities in Zn3+S, Zn4+S, and Zn5+S treatment groups were significantly higher than those in the CK. Furthermore, the CA activity (Figure 3d) in the Zn4+S group also surpassed the level observed in the CK group.

### 3.3. Effects of ZnO NPs on Superoxide Anion (SA), Malondialdehyde (MDA), Electrolyte Leakage (EL) and Membrane Stability Index (MSI) of G. uralensis Under NaCl Stress

**Figure 4:**
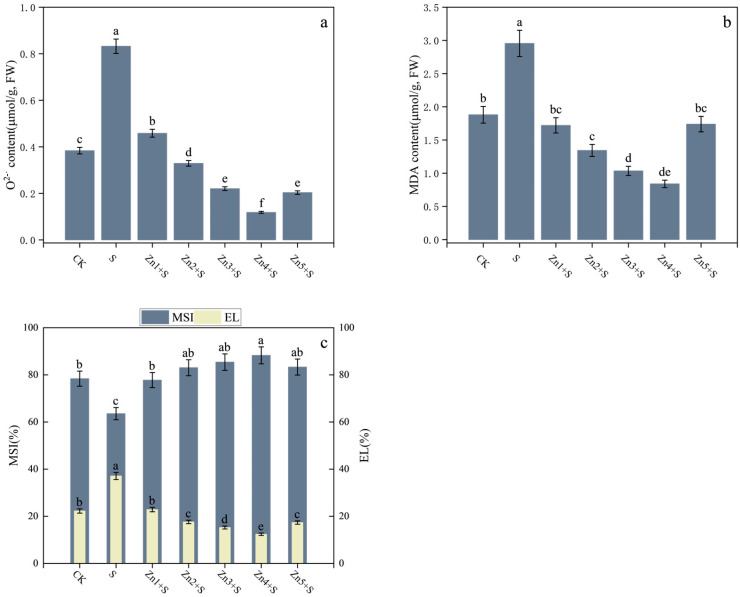
Effects of ZnO NPs application on the content of osmotic adjustment substances in the leaves of *G. uralensis* under salt stress. (**a**) superoxide anion content; (**b**) MDA content; (**c**) electrolytic leakage (EL) and membrane stability index (MSI). FW denotes fresh weight basis. Bars represent mean ± SD (n = 3). Different lowercase letters indicate statistically significant differences among treatments at *p* < 0.05 according to Duncan’s multiple range test.

NaCl (S treatment) triggered a strong oxidative stress on *G. uralensis*. Compared to the CK, S treatment increased superoxide anion, MDA, and EL levels by 117%, 57%, and 67%, respectively, while reducing the MSI by 19% (Figure 4). In contrast, the combined treatment of 30 mg/kg ZnO NPs with NaCl (Zn4+S) reduced superoxide anion, MDA, and EL levels by 86%, 72%, and 67%, respectively, compared to the S treatment, while increasing MSI by 39%. These findings demonstrate that ZnO NPs application, particularly at 30 mg/kg, significantly mitigates oxidative stress in NaCl-stressed *G. uralensis*. The enhanced MSI levels indicate improved maintenance of cellular membrane integrity under saline conditions, thereby strengthening the plant’s salt stress tolerance capacity.

### 3.4. Effects of ZnO NPs on Soluble Protein, Proline, and Soluble Sugar Concentration of NaCl-Stressed G. uralensis

**Figure 5:**
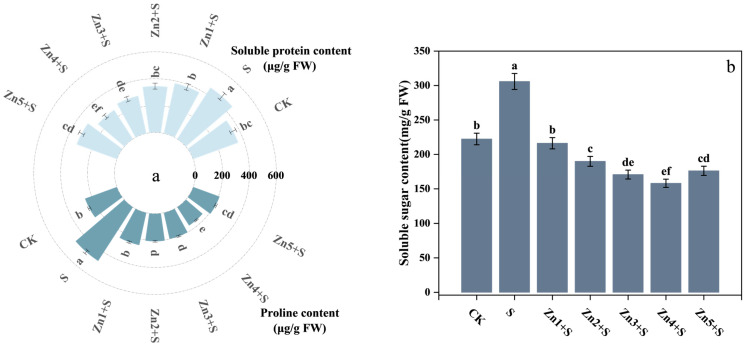
Effects of ZnO NPs application on the concentration of osmotic adjustment substances in the leaves of *G. uralensis* under salt stress. (**a**) Soluble protein and proline content; (**b**) soluble sugar content. FW denotes fresh weight basis. Bars represent mean ± SD (n = 3). Different lowercase letters indicate statistically significant differences among treatments at *p* < 0.05 according to Duncan’s multiple range test.

We found that NaCl (S treatment) induced obvious osmotic stress in *G. uralensis*, as evidenced by substantial increases in soluble protein, proline, and soluble sugar concentration in leaves (Figure 5a,b). Concentration of soluble protein, proline, and soluble sugar in S treatment was higher than those of the CK by 28%, 89%, and 38%. All combined treatments of ZnO NPs with NaCl significantly reduced these osmotic adjustment substances compared to NaCl treatment alone. Notably, the 30 mg/kg ZnO NPs + NaCl treatment exhibited the most pronounced reductions, decreasing soluble protein, proline, and soluble sugar contents by 44%, 66%, and 48%.

### 3.5. Effects of ZnO NPs on the Contents of Glycyrrhizic Acid, Liquiritin, and Total Flavonoids in the Roots of NaCl-Stressed G. uralensis

**Figure 6:**
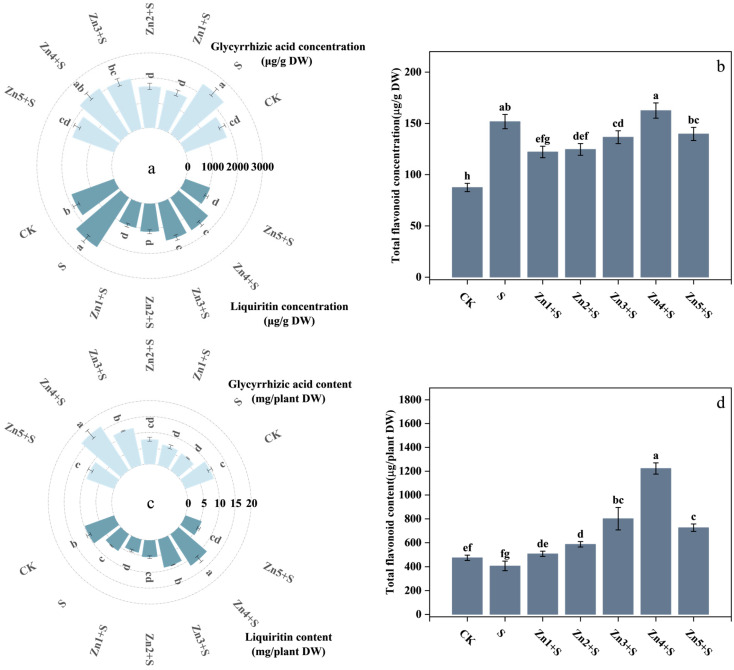
Effects of ZnO NPs application on medicinal compound contents in *G. uralensis* roots under salt stress. (**a**) Concentrations of glycyrrhizic acid (GA) and liquiritin (Li); (**b**) total flavonoid (TF) concentration; (**c**) GA and Li contents per taproot; (**d**) TF content per taproot. DW denotes dry weight basis. Bars represent mean ± SD (n = 3). Different lowercase letters indicate statistically significant differences among treatments at *p* < 0.05 according to Duncan’s multiple range test.

Although the S treatment exhibited 35%, 30%, and 73% increases in root concentrations of glycyrrhizic acid, liquiritin, and total flavonoids compared to the CK (Figure 6a,b), this treatment strongly inhibited root growth and reduced root biomass (Figure 1a), resulting in 34%, 36%, and 14% decreases in the total contents of these bioactive compounds per plant, respectively. However, the combined application of 30 mg/kg ZnO NPs with NaCl (Zn4+S treatment) not only significantly enhanced root biomass (Figure 1a) but also substantially promoted concentrations of glycyrrhizic acid and total flavonoids by 23% and 85.7% compared to CK. Consequently, the Zn4+S treatment elevated per-plant contents of glycyrrhizic acid, liquiritin, and total flavonoids by 158%, 87%, and 201%, respectively, compared to the S treatment alone.

### 3.6. Principle Component Analysis (PCA)

To evaluate the ameliorative effects of different ZnO NPs dosages on salt stress and their enhancement effects on medicinal organ yield and quality in *G. uralensis*, principal component analysis (PCA) was conducted using SPSS 23.0 software to comprehensively assess multiple measured parameters across treatments. Both the initial eigenvalues of two principal components exceeded 1, with a cumulative contribution rate reaching 88.339% (Table 4). The first principal component (PC1) accounted for 74.007% of the variance, primarily driven by growth-related parameters: RD (0.978), BD (0.969), SDW (0.967), H (0.967), RDW (0.939), and TRL (0.961). The second principal component (PC2) contributed 14.332% of the variance, predominantly associated with secondary metabolite synthesis and osmotic regulation indicators: Li (0.894), GA (0.707), and Pro (0.561).

According to the matrix in Table 4, the scoring coefficients (*Y*_1_, *Y*_2_) of the two principal components and the comprehensive score (*Y*) were calculated. The calculation formula is as follows:

*Y*_1_ = 0.234 × *Z*_1_ + 0.228 × *Z*_2_ + 0.235 × *Z*_3_ + 0.237 × *Z*_4_ + 0.234 × *Z*_5_ + 0.233 × *Z*_6_ + 0.183 × *Z*_7_ + 0.208 × *Z*_8_ + 0.208 × *Z*_9_ + 0.218 × *Z*_10_ + 0.190 × *Z*_11_ + 0.229 × *Z*_12_ + 0.229 × *Z*_13_ − 0.217 × *Z*_14_ − 0.218 × *Z*_15_ − 0.215 × *Z*_16_ + 0.2_15_ × *Z*_17_ − 0.237 × *Z*_18_ − 0.227 × *Z*_19_ − 0.187 × *Z*_20_ + 0.145 × *Z*_21_ + 0.080 × *Z*_22_ + 0.081 × *Z*_23_

*Y*_2_ = −0.059 × *Z*_1_ + 0.001 × *Z*_2_ − 0.005 × *Z*_3_ + 0.087 × *Z*_4_ − 0.039 × *Z*_5_ + 0.088 × *Z*_6_ + 0.295 × *Z*_7_ + 0.143 × *Z*_8_ + 0.211 × *Z*_9_ + 0.130 × *Z*_10_ + 0.148 × *Z*_11_ − 0.103 × *Z*_12_ + 0.028 × *Z*_13_ + 0.228 × *Z*_14_ + 0.188 × *Z*_15_ + 0.231 × *Z*_16_ − 0.231 × *Z*_17_ + 0.050 × *Z*_18_ + 0.176 × *Z*_19_ + 0.309 × *Z*_20_ + 0.390 × *Z*_21_ + 0.492 × *Z*_22_ + 0.244 × *Z*_23_

*Y* = 74.007% × *Y*_1_ + 14.332% × *Y*_2_

In the equations, *Y*_1_ and *Y*_2_ represent the scores of principal component 1 and 2, respectively; *Y* denotes the comprehensive score; *Z*_1_–*Z*_27_ correspond to the standardized values of variables SDW, RDW, BD, RD, H, TRL, RAD, TRSA, TRV, SOD, POD, CAT, CA, SA, MDA, EL, MSI, SP, SS, Pro, GA, Li, TF (processed via the *Z*-score method) as listed in Table 5.

Based on the comprehensive scores derived from principal component analysis (PCA) (Table 6), the application of 30 mg/kg ZnO NPs under NaCl stress condition most significantly enhanced *G. uralensis* growth while concurrently improving yield and quality of its medicinal organ, and the salt tolerance capacity of the licorice.

### 3.7. Pearson Correlation Analysis

**Figure 7:**
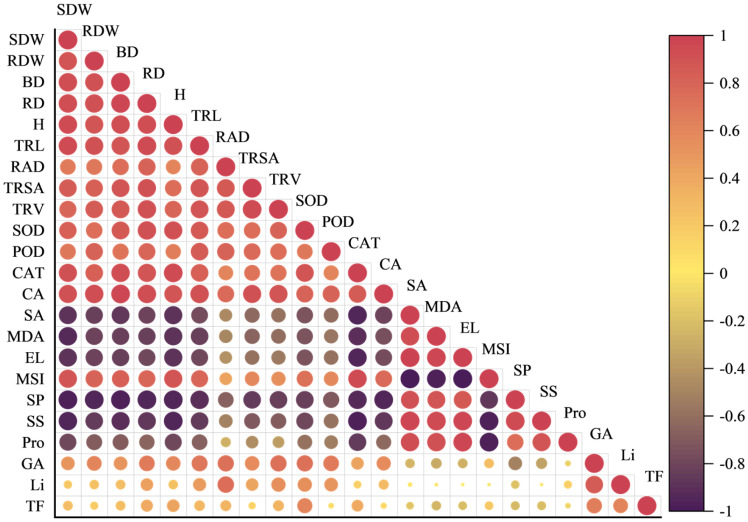
The correlation matrix between 23 parameters. The positive correlation is expressed in red, and the negative correlation is expressed in blue. The deeper the color, the higher the correlation between the two variables. SDW, shoot dry weight; RDW, root dry weight; BD, basal diameter; RD, root diameter; H, height; TRL, total root length; RAD, roots average diameter; TRSA, total root surface area; TRV, total root volume; SOD, superoxide dismutase; POD, peroxidase; CAT, catalase; CA, carbonic anhydrase; SA, superoxide anion; MDA, malondialdehyde; EL, electrolytic leakage; MSI, membrane stability index; SP, soluble protein; SS, soluble sugar; Pro, proline; GA, glycyrrhizic acid; Li, liquiritin; TF: total flavonoids.

To comprehensively investigate the effects of ZnO NPs application on the plant, Pearson correlation analysis was conducted on 23 traits. The correlation coefficient matrix (Figure 7) revealed strong positive correlations between yield-related traits (root diameter and root dry weight) and root morphological parameters (total root length, total root surface area, and total root volume). Shoot dry weight, stem basal diameter, plant height, and total root length exhibited strong positive correlations, indicating that ZnO NPs application positively influenced the coordinated development of shoot and root. CAT activity was strongly negatively correlated with superoxide anion content, MDA levels, and electrolyte leakage, while positively correlating with MSI, highlighting CAT’s crucial role in ROS scavenging and membrane system stabilization. CA activity negatively correlated with soluble protein content. Quality-related traits (glycyrrhizic acid, liquiritin, and total flavonoid contents) positively correlated with root diameter, root average diameter, and total root volume, demonstrating ZnO NPs’ beneficial effects on both root growth and medicinal compound accumulation. In summary, correlation analysis indicated that ZnO NPs application under NaCl stress enhanced antioxidant capacity through increased CAT activity, promoted yield of medicinal organ via improved root architecture and development, and facilitated positive interactions between shoot and root growth. Furthermore, ZnO NPs application simultaneously enhanced root growth and its medicinal quality in *G. uralensis*.

## 4. Discussion

Consistent with numerous previous studies [37,38,39], our findings demonstrated that NaCl stress significantly inhibited root growth in *G. uralensis*, leading to substantial reduction in yield of its medicinal organ. The growth inhibition under salt stress may be regulated by endogenous hormones, particularly through abscisic acid (ABA) synthesis, which suppresses plant growth and accelerates senescence [19]. Compared to conventional Zn fertilizers, ZnO NPs have been shown to accelerate developmental processes in wheat (*Triticum aestivum* L.) [40] and promote biomass accumulation in spinach (*Spinacia oleracea* cv. Chunqiu Daye) at low concentration [41], potentially due to their higher solubility [42]. Elsherif et al. [43] and Francis et al. [44] found that the appropriate amount ZnO NPs application significantly increased biomass in fenugreek (*Trigonella foenum-graecum* L.) and *Amaranthus hybridus* seedlings, indicating that ZnO NPs as nano-fertilizer can promote plant growth more effectively than traditional fertilizer. Dang et al. reported that ZnO NPs improved salt tolerance of rice (*Oryza sativa*), significantly enhancing its biomass, height, and root length [45]. Our study revealed that ZnO NPs application significantly alleviates NaCl-induced growth inhibition in *G. uralensis*. PCA results indicated that the Zn4+S treatment group exhibited optimal ameliorative effects, simultaneously enhancing salt tolerance and maximizing vegetative growth. Pearson correlation analysis showed that ZnO NPs may increase root yield by enhancing photosynthetic efficiency in leaves, while potentially stimulating indole-3-acetic acid (IAA) synthesis [46] to improve vegetative growth of the plant. Notably, even under 160 mM NaCl stress, several growth parameters in *G. uralensis* treated with 30 mg/kg ZnO NPs exceeded those of the CK, further confirming the beneficial role of ZnO NPs in enhancing salt tolerance of the licorice.

During long-term evolution, plants have developed an efficient defense system against environmental stresses, comprising antioxidant enzymes (e.g., SOD, POD, CAT) and non-enzymatic components [47]. This system effectively scavenges reactive oxygen species (ROS) to mitigate oxidative stress [48,49]. Carbonic anhydrase (CA), which catalyzes the reversible hydration of CO_2_ (CO_2_ + H_2_O ⇌ HCO_3_^−^ + H^+^), serves as a biochemical indicator of photosynthetic efficiency [50], though its activity is strongly dependent on Zn availability. Our findings reveal that 160 mM NaCl stress significantly reduced SOD, POD, CAT, and CA activities in *G. uralensis* leaves, indicating severe oxidative stress [51]. Compared to the CK, NaCl-treated plants exhibited significant increases in superoxide anion, malondialdehyde (MDA), and electrolyte leakage (EL) levels, alongside a decrease in membrane stability index (MSI), demonstrating accelerated lipid peroxidation and compromised membrane integrity. Under stress conditions, excessive ROS oxidizes fatty acids in biomembranes, generating MDA—a key biomarker for assessing oxidative damage severity [52]. Membrane peroxidation increases permeability, reduces stability, and elevates electrolyte leakage through cytoplasmic efflux. Nanoparticle applications enhance plant stress resilience more effectively than bulk materials. For instance, calcium oxide nanoparticles (CaO NPs) significantly improved antioxidant enzyme activities (SOD, CAT, POD, and APX) in alfalfa (*Medicago sativa* L.) compared to conventional CaO [53]. Similarly, ZnO NPs upregulated CAT gene expression in soybean (*Glycine max* L.) more potently than regular ZnO [54]. In our study, the Zn4+S treatment (30 mg/kg ZnO NPs + NaCl) reduced superoxide anion, MDA, and EL levels, while increasing MSI, demonstrating enhanced antioxidant capacity through ZnO NPs application [55]. This improvement likely stems from Zn’s critical role as a cofactor in antioxidant enzyme biosynthesis and protein stabilization [56], thereby boosting stress tolerance [57,58]. PCA results further confirmed that Zn4 treatment under 160 mM NaCl stress achieved the highest SOD, POD, CAT, and CA activities highlighting ZnO NPs’ dual benefits in ROS scavenging and photosynthetic optimization.

Zinc plays an important role in maintaining plant redox homeostasis [59]. This regulatory function may stem from Zn’s incorporation into the Cu/Zn-SOD enzyme, which catalyzes the dismutation of superoxide radicals (O_2_^−^ → H_2_O_2_ + O_2_), thereby controlling reactive oxygen species (ROS) generation. Furthermore, Zn application reduces thiobarbituric acid reactive substances (TBARS) content—a lipid peroxidation biomarker—in stressed plants [60]. Additionally, Zn enhances indole-derived auxin biosynthesis. As demonstrated by Elsheery et al. [61], auxins exhibit multifunctional stress mitigation effects: ROS scavenging, facilitating nutrient translocation, inhibiting Na^+^ uptaking, promoting cell division, maintaining membrane stability, and functional phospholipid accumulation, suggests that Zn may promote plant growth and reduce the degree of lipid peroxidation of cell membrane under salt stress by regulating endogenous hormone levels. Pearson correlation analysis showed that CAT activity was negatively correlated with superoxide anion, MDA, electrolyte leakage rate, and positively correlated with MSI, indicating that the treatment of ZnO NPs significantly increased CAT activity in *G. uralensis* and effectively maintained the stability of its cell membrane system. The above results showed that ZnO NPs could improve the salt tolerance of the licorice by increasing the activity of enzymes, thus promoting the yield of its medicinal organ under NaCl stress.

This study investigated the effects of NaCl stress on osmotic balance in *G. uralensis* and the regulatory role of exogenous ZnO NPs in osmotic adjustment. The osmolytes, such as soluble proteins, proline, and soluble sugars, serve to maintain cellular water potential and enhance stress tolerance of a plant. However, excessive accumulation of these compounds may reflect metabolic strain under severe saline conditions. Our results showed that NaCl stress significantly increased leaf soluble protein, proline, and soluble sugar contents. While these osmolytes help stabilize cellular osmotic pressure, their synthesis likely diverted substantial energy and carbon resources, thereby limiting nutrient allocation for vegetative growth [62]—a common trade-off in plant osmotic stress responses [63]. Notably, ZnO NPs demonstrated superior efficacy in mitigating abiotic stress compared to bulk ZnO, as evidenced by lower proline accumulation in soybean (*Glycine max* L.) under ZnO NPs treatment compared to bulk ZnO treatment [54]. Principal component analysis (PCA) revealed that the application of 30 mg/kg ZnO NPs under NaCl stress (Zn4+S) significantly reduced the content of these osmotic adjustment substances compared to NaCl treatment alone, potentially through Zn^2+^-mediated regulation of ion homeostasis. Correlation analysis identified a negative relationship between carbonic anhydrase (CA) activity and soluble protein content, suggesting that ZnO NPs-supplied Zn^2+^ may enhance enzymatic coordination of osmotic adjustment. Supporting this, Mushtaq et al. [64] demonstrated that the appropriate amount Zn supplementation increased proline metabolism-related enzyme activities. Furthermore, ZnO NPs likely alleviated osmotic stress by reducing Na^+^ uptake, thereby minimizing ionic imbalance [65]. Critically, the combined ZnO NPs + NaCl treatment decreased soluble protein, proline, and soluble sugar contents, compared to NaCl treatment alone. These findings confirm ZnO NPs’ capacity to alleviate osmotic adjustment imbalance caused by NaCl stress.

The medicinal quality of *G. uralensis* is primarily determined by the contents of glycyrrhizic acid, liquiritin, and flavonoids in its root [66,67,68]. Under 160 mM NaCl stress, the concentrations of these bioactive compounds in the root significantly increased, suggesting their accumulation probably serves as a stress adaptation mechanism. Similar patterns were observed in salt-stressed tomato (*Solanum lycopersicum* L.), where terpenoid concentrations positively correlated with salinity levels [69]. Proteomic analyses further confirmed NaCl stress upregulates key enzymes in glycyrrhizic acid and liquiritin biosynthesis pathways in *G. uralensis* roots [70]. Salt stress-induced secondary metabolite accumulation—a universal plant adaptive strategy—enhances antioxidant, anti-inflammatory, and antimicrobial activities critical for stress resilience [71]. However, despite elevated metabolite concentrations, NaCl stress reduced root biomass, leading to decreases in per-plant glycyrrhizic acid, liquiritin, and flavonoid contents compared to CK. This reflects a resource allocation trade-off: while prioritizing defense compound synthesis [72], a plant sacrifices growth-related resource investment. The addition of ZnO NPs under NaCl stress not only significantly promoted the growth of *G. uralensis* roots and increased the biomass of roots, but also significantly increased the content of its medicinal components. Especially under the condition of NaCl stress, after adding 30 mg/kg ZnO NPs (Zn4+S treatment), the content of glycyrrhizic acid, liquiritin and total flavonoids in the roots of *G. uralensis* increased most obviously, and their content reached 2.6 times, 1.9 times and 3 times as much as those of the S group, respectively. The content even exceeded the CK group, that is, the yield of medicinal organs and the content of medicinal components were much higher than those in non-salinized environment. Correlation analysis revealed positive relationships between contents of the medicinal compounds and root morphological parameters and growth parameters, confirming ZnO NPs’ dual optimization of officinal quality and yield of the roots. The enhanced taproot diameter particularly facilitated metabolite storage capacity. These findings demonstrated that optimal ZnO NPs supplementation (30 mg/kg) under saline conditions simultaneously improves medicinal yield (through biomass enhancement) and quality (via phytochemical enrichment), even surpassing non-stressed cultivation outcomes. This nano-enabled strategy effectively decouples the stress defense-growth trade-off, positioning ZnO NPs as a transformative agent for medicinal plant cultivation in saline soil.

Previous research demonstrates nanoparticle-enhanced stress resilience across diverse crops, iron nanoparticles (Fe NPs) improved drought tolerance in spinach (*Spinacia oleracea* L.) [73], while comparative analysis of four nanoparticles (TiO_2_, SiO_2_, ZnO, Fe_2_O_3_) revealed ZnO NPs most significantly enhanced salt tolerance in flax (*Linum usitatissimum* L.) under equivalent experimental conditions [74]. Similarly, silver nanoparticles (Ag NPs) mitigated combined salt-drought stress in quinoa (*Chenopodium quinoa*) [75]. Collectively, extant literature substantiates the exceptional potential of nanoparticles for multidimensional crop enhancement, thus providing the foundational impetus for this investigation into their efficacy in *G. uralensis* under saline stress conditions.

There are challenges in large-scale application and commercialization of nano-fertilizers. Large-scale synthesis of uniform ZnO NPs remains energy-intensive, with current methods incurring high production costs compared to conventional fertilizers. Long-term ecological impacts of nanoparticle accumulation in soil–plant systems, particularly under repeated application, require further assessments [76,77]. Standardized protocols for nanofertilizer toxicity evaluation and field-scale certification are lacking globally, hindering commercialization. Nowadays, people are also actively exploring these challenges. Utilizing plant extract-mediated or microbial synthesis of ZnO NPs could reduce costs and enhance biocompatibility [78,79]. Encapsulating NPs in biodegradable polymers may minimize environmental leakage while improving nutrient uptake efficiency [80]. Establishing international consortia to unify safety standards and methods, as exemplified by applying the (Q) SAR model to assess the risk of nanomaterials [81].

## 5. Conclusions

The addition of 30 mg/kg ZnO significantly improved the salt tolerance of the licorice under the NaCl stress, significantly increasing the yield and quality of its medicinal organ. The specific performance was that the content of osmotic adjustment substances decreased, the activities of antioxidant enzyme increased significantly, the membrane stability increased, and the activity of CA increased. Root diameter and root biomass increased significantly, and the content of medicinal substances in the roots was significantly increased. In summary, the results of this study showed that exogenous ZnO NPs had a significant effect on improving the salt tolerance of *G. uralensis* and the yield and quality of its medicinal organ, and the effect was the best when 30 mg/kg ZnO NPs were added. Future research will systematically investigate the temporal dynamics and mechanistic pathways of ZnO NPs in enhancing *Glycyrrhiza uralensis* resilience under saline conditions. This includes optimizing application protocols through growth-stage-specific dosing trials and evaluating diverse application strategies (e.g., foliar spraying). Mechanistic analyses will integrate molecular profiling, rhizosphere microbiome dynamics via metagenomic sequencing, and ion homeostasis regulation to elucidate the synergistic interactions governing ZnO NP-mediated improvements in salt tolerance, biomass yield, and phytochemical quality.

## Figures and Tables

**Table 1:** Properties of the sandy soil.

Total Nitrogen g/kg	Total Phosphorus g/kg	Total Potassium g/kg	Available N mg/kg	Available P mg/kg	Available K mg/kg	Organic Matter g/kg
0.315	0.131	5.47	52.59	5.23	50.04	6.64

**Table 2:** Experimental treatments.

Treatments Abbreviations	NaCl Concentration (mM)	ZnO NPs Concentration (mg/kg)
CK	0	0
S	160	0
Zn1+S	160	10
Zn2+S	160	15
Zn3+S	160	20
Zn4+S	160	30
Zn5+S	160	40

Tips: S: NaCl, Zn: ZnO NPs.

**Table 3:** Regression equations, correlation coefficients of liquiritin and glycyrrhizic acid.

Compounds	Retention Time (min)	Regression Equation	Correlation Coefficients (R^2^)	Wave Length (nm)
Li	5.723	Y = 9.12592X + 0.258855	0.9999	276
GA	6.829	Y = 3.68535X − 4.47923	0.9999	254

**Table 4:** Principal component analysis load matrix.

Parameters	PC1	PC2
SDW	0.967	−0.107
RDW	0.939	0.002
BD	0.969	−0.010
RD	0.978	0.158
H	0.967	−0.071
TRL	0.961	0.160
RAD	0.754	0.536
TRSA	0.860	0.259
TRV	0.859	0.383
SOD	0.901	0.236
POD	0.783	0.269
CAT	0.946	−0.188
CA	0.943	0.051
SA	−0.897	0.414
MDA	−0.898	0.342
EL	−0.888	0.420
MSI	0.888	−0.420
SP	−0.977	0.091
SS	−0.937	0.320
Pro	−0.772	0.561
GA	0.600	0.707
Li	0.329	0.894
TF	0.336	0.443
Eigen values	17.022	3.296
Proportion of variance (%)	74.007	14.332
Cumulative variance (%)	74.007	88.339

**Table 5:** Standardized values of each parameters of *G. uralensis* were applied with ZnO NPs under NaCl stress.

Parameters	Treatments					
	S	S+Zn1	S+Zn2	S+Zn3	S+Zn4	S+Zn5
SDW	−2.090	−0.673	−0.325	0.070	0.574	−0.649
RDW	−1.828	−1.064	−0.776	−0.186	0.678	−0.525
BD	−1.743	−1.383	−0.733	−0.157	0.567	−0.139
RD	−1.458	−1.208	−0.834	−0.043	0.727	−0.549
H	−1.879	−0.844	−0.298	−0.028	0.651	0.033
TRL	−1.646	−0.824	−0.429	−0.089	0.410	−0.999
RAD	−0.332	−1.654	−0.778	−0.363	0.386	−1.069
TRSA	−1.082	−1.199	−0.767	−0.690	0.486	−1.283
TRV	−0.717	−1.105	−0.962	−0.920	0.477	−0.903
SOD	−1.124	−1.054	−0.670	−0.050	0.522	0.246
POD	−1.400	−1.280	−0.726	−0.293	0.285	−0.606
CAT	−2.090	−1.094	−0.159	0.101	0.796	0.392
CA	−1.749	−1.258	−0.749	−0.416	0.045	−0.379
SA	2.644	0.827	0.200	−0.329	−0.825	−0.412
MDA	2.476	0.475	−0.137	−0.636	−0.954	0.505
EL	2.675	0.629	−0.124	−0.465	−0.874	−0.163
MSI	−2.675	−0.629	0.124	0.465	0.874	0.163
SP	1.986	1.086	0.631	−0.095	−0.446	0.312
SS	2.470	0.681	0.157	−0.225	−0.478	−0.117
Pro	2.956	0.149	−0.352	−0.427	−0.869	−0.308
GA	0.443	−0.942	−0.820	−0.146	0.074	−0.611
Li	1.282	−0.912	−0.742	−0.057	−0.041	−0.870
TF	0.810	−0.302	−0.208	0.239	1.214	0.358

**Table 6:** Comprehensive evaluation scores.

Treatments	*Y*_1_	*Y*_2_	Composite Scores *Y*	Ranging
CK	−2.106	0.572	−1.671	4
S	−7.927	4.098	−5.976	7
S+Zn1	−4.423	−1.341	−3.923	6
S+Zn2	−2.061	−1.641	−1.993	5
S+Zn3	−0.083	−1.098	−0.248	2
S+Zn4	2.720	−0.491	2.199	1
S+Zn5	−1.437	−1.651	−1.472	3

## Data Availability

Data are contained within the article.
